# Revisiting the Continuum Hypothesis: Toward an In-Depth Exploration of Executive Functions in Korsakoff Syndrome

**DOI:** 10.3389/fnhum.2014.00498

**Published:** 2014-07-04

**Authors:** Mélanie Brion, Anne-Lise Pitel, Hélène Beaunieux, Pierre Maurage

**Affiliations:** ^1^Laboratory for Experimental Psychopathology, Institute of Psychology, Université Catholique de Louvain, Louvain-la-Neuve, Belgium; ^2^INSERM, École Pratique des Hautes Études, Université de Caen-Basse Normandie, Unité U1077, GIP Cyceron, CHU Caen, Caen, France

**Keywords:** alcohol-dependence, executive functions, inhibition, Korsakoff syndrome

## Abstract

Korsakoff syndrome (KS) is a neurological state mostly caused by alcohol-dependence and leading to disproportionate episodic memory deficits. KS patients present more severe anterograde amnesia than Alcohol-Dependent Subjects (ADS), which led to the continuum hypothesis postulating a progressive increase in brain and cognitive damages during the evolution from ADS to KS. This hypothesis has been extensively examined for memory but is still debated for other abilities, notably executive functions (EF). EF have up to now been explored by unspecific tasks in KS, and few studies explored their interactions with memory. Exploring EF in KS by specific tasks based on current EF models could thus renew the exploration of the continuum hypothesis. This paper will propose a research program aiming at: (1) clarifying the extent of executive dysfunctions in KS by tasks focusing on specific EF subcomponents; (2) determining the differential EF deficits in ADS and KS; (3) exploring EF-memory interactions in KS with innovative tasks. At the fundamental level, this exploration will test the continuum hypothesis beyond memory. At the clinical level, it will propose new rehabilitation tools focusing on the EF specifically impaired in KS.

## Introduction

The negative consequences of chronic excessive alcohol consumption on health are well established, alcohol-dependent subjects (ADS) presenting impairments in several body systems (Rehm et al., 2009; Bühler and Mann, 2011). Alcohol misuse can also lead to nutritional problems increasing the risk of thiamine deficiency (Victor et al., 1971; Lough, 2012). Repeated thiamine deprivation can provoke cerebral disorders such as the Wernicke’s encephalopathy, a medical emergency with lethal risk (Thomson and Marshall, 2006) potentially progressing toward Korsakoff syndrome (KS). KS is a neurological complication of alcohol-dependence (Victor et al., 1971; Isenberg-Grzeda et al., 2012) combining alcohol neurotoxicity and thiamine deficiency (Brand, 2007; Pitel et al., 2008; Fama et al., 2012). The cardinal KS symptom is a permanent anterograde and retrograde amnesia, which has been widely explored. However, other cognitive impairments related to KS, and notably executive functions (EF) known to be highly impaired in alcohol-dependence, have been less explored.

After reviewing earlier neuropsychological explorations of KS, this paper will underline the importance of further exploring EF in this pathology. A research program will be proposed, with three crucial aims: (1) clarifying the extent of executive dysfunctions in KS; (2) determining the differential deficits across specific EF in ADS and KS; (3) exploring the interactions between EF and memory impairments in KS. This thorough exploration might lead to a new model of cognitive impairments in KS, which will have crucial implications at fundamental (i.e., revaluation of the continuum hypothesis proposing the continuity in the impairments between ADS and KS) and clinical (i.e., new neuropsychological rehabilitation perspectives) levels.

## What Do We Know about Memory Deficits in KS?

Severe retrograde and anterograde amnesia is the most explored symptom in KS (Brand et al., 2009; Fama et al., 2012; Kessels and Kopelman, 2012; Pitel et al., 2012; Sullivan and Fama, 2012). Indeed, thiamine deficiency affects diencephalic and limbic structures (Borsutzky et al., 2008; Labudda et al., 2008; Brand et al., 2009), leading to episodic memory disorders (Pitel et al., 2011). KS notably present abnormalities in the hippocampi, mammillary bodies, and thalamic nuclei (Pitel et al., 2012), these regions being involved in episodic memory (Brand et al., 2009; Harper, 2009; Kopelman et al., 2009; Sullivan and Pfefferbaum, 2009; Kril and Harper, 2012). Episodic memory is a long-term memory subcomponent gathering features related to specific events, situations, and experiences, and involving the encoding, storage, and retrieval of the event and its spatio-temporal context (Tulving, 2001). Episodic memory system also encompass autonoetic consciousness, the ability to time traveling into one’s own recollections (Wheeler et al., 1997). Therefore, many studies (Brand, 2007; d’Ydewalle and Van Damme, 2007; Pitel et al., 2008) separately investigated each episodic memory components and confirmed that KS have deficits for episodic encoding and retrieval, impaired contextual memory, and altered autonoetic consciousness. Therefore, KS clearly present lower performance than controls and AD in episodic memory, this impairment being associated with reduced thalamic and frontal volumes (Shimamura et al., 1988).

Other memory systems are also impaired in KS, particularly: (1) autobiographical memory, including general knowledge, semantic information, and personal events (Race and Verfaellie, 2012); (2) implicit learning, tested with motor (Hayes et al., 2012), and cognitive (Beaunieux et al., 2013) skill learning. Conversely, the working memory slave systems (i.e., phonological loop and visuospatial sketchpad; Baddeley et al., 1996) involved in the encoding phase (Van Geldorp et al., 2012) appear relatively preserved in KS (Pitel et al., 2008).

Beside memory systems, abilities simultaneously involving memory and other cognitive functions have also been partly investigated. Confabulations, namely unintentional and incongruous verbal production (Dalla Barba and Decaix, 2009; Kessels and Kopelman, 2012) have been described in KS (Borsutzky et al., 2008; Bouzerda-Wahlen et al., 2013) and are usually interpreted as related to memory dysfunction. However, while involving memory, they might also be related to other cognitive abilities, notably EF (Metcalf et al., 2007). Indeed, spontaneous confabulations theories emphasized the role of frontal cortices and EF dysfunction in their occurrence (Metcalf et al., 2007; Dalla Barba and Decaix, 2009). Other cognitive abilities simultaneously involving memory and EF have also been explored, particularly: (1) metamemory, impaired in ADS (Le Berre et al., 2010) and modulated by EF impairments; (2) cognitive procedural learning, altered in KS and ADS (Beaunieux et al., 2013) and also relying on EF. The memory impairments described in KS thus seem to be at least partly related to EF impairments.

## What Do We Know about Executive Functioning in KS?

Beyond memory, the description of executive impairments in KS led to the proposal that KS should be reconsidered as a frontal lobe pathology (Van Oort and Kessels, 2009; Jung et al., 2012; Oscar-Berman, 2012; Maharasingam et al., 2013). Frontal lobes are crucial for EF and, as they are highly vulnerable to alcohol neurotoxicity (Moselhy Hamdy, 2001; Reed et al., 2003; Oscar-Berman, 2012; Pitel et al., 2012; Maharasingam et al., 2013), alcohol-dependence leads to largely explored EF deficits (Stavro et al., 2013). EF impairments have also been explored in KS, but their understanding should be renewed as EF contribution to amnesia remains debated (Kessels and Kopelman, 2012; Oscar-Berman, 2012).

Two contradictory proposals indeed exist. First, as alcohol neurotoxicity mostly affects frontal lobes (Fujiwara et al., 2008) while thiamine deficiency affects diencephalic regions (Jacobson and Lishman, 1990; Brokate et al., 2003; Brand, 2007; Fama et al., 2012), ADS, and KS might be characterized by similar EF impairments (due to alcohol effects on frontal regions), the difference being found for memory (increased in KS due to thiamine deficiency). Neuropsychological investigations indeed showed parallel EF impairments in ADS and KS, with more severe episodic memory deficits in KS (Pitel et al., 2008). Second and conversely, the continuum hypothesis (Butters and Brandt, 1985; Pitel et al., 2008; Sullivan and Pfefferbaum, 2009) postulates continuity in cognitive impairments between ADS and KS, KS being centrally characterized by a global worsening of alcohol-dependence deficits which cannot be fully accounted by differences in drinking history (Pitel et al., 2008), contrary to early statements (Ryback, 1971). KS would thus present stronger deficits than ADS for memory, but also for EF (Oscar-Berman et al., 2004; Jung et al., 2012). However, while EF have been studied in KS, most studies focus on memory and the exploration of EF deficits in KS should be deepened. In short, it is now accepted that KS have disproportionate memory impairment across episodic memory tasks whereas there is no clear-cut opinion about (dis)continuity between ADS and KS for other cognitive functions. There is thus insufficient evidence to determine whether there is a continuum of EF impairment from ADS to KS.

**Figure 1:**
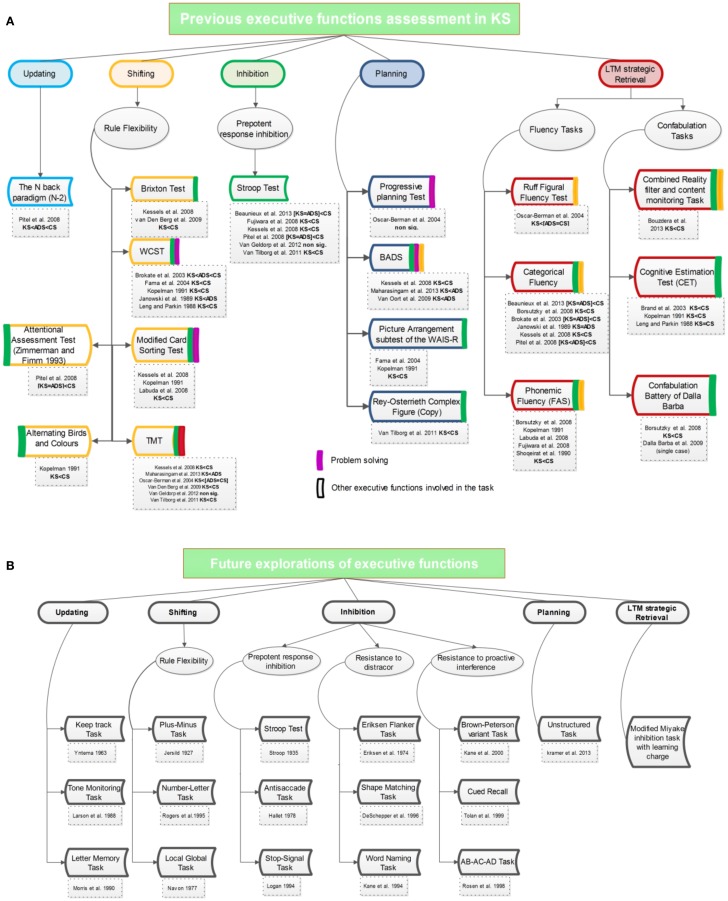
**(A)** Overview of multifaceted tasks previously used for EF assessment in KS. Tasks are classified according to their central EF subcomponent. Color rectangles at the right of each task underline the other EF subcomponents involved in this task. Purple rectangles indicate the overlap between EF subcomponents and problem solving (a complex and transversal EF involved in several tasks). **(B)** Overview of the EF assessment program proposed by Miyake et al. (2000) and Friedman and Miyake (2004).

Studies that investigated EF in KS used a wide variety of unspecific tasks simultaneously tapping into different EF without being theoretically grounded. An inventory of EF tasks used in KS is presented in Figure 1A. Studies were classified according to Miyake et al. (2000) categorization. Indeed, while EF organization remains questionable (Fournier-Vicente et al., 2008; Hull et al., 2008; Adrover-Roig et al., 2012), this multi-factorial model clearly subdivides EF into three basic factors, to which two complex factors were recently added (Fournier-Vicente et al., 2008; Adrover-Roig et al., 2012): (1) shifting, the ability to transfer cognitive resources across tasks; (2) updating, the ability to replace irrelevant information by pertinent new ones; (3) inhibition, the control ability preventing a non-pertinent automatic or dominant response to occur; (4) planning, the self-regulation based on a strategic elaboration of the successive stages in non-routine situations; (5) long-term memory strategic retrieval, the selection of correct information in memory.

As shown in Figure 1, early findings (e.g., Janowsky et al., 1989; Kopelman, 1991) showed that KS cognitive deficits are partly attributable to frontal lobe dysfunctions. A comparison between Alzheimer and KS patients (Kopelman, 1991) clearly illustrated the presence of frontal impairment in KS and its influence on retrograde amnesia. A comparison between patients with frontal lesion, amnesia, and KS also demonstrated that frontal lobe damage is a specific cognitive pattern for KS compared to other types of amnesia (Janowsky et al., 1989). More recent findings demonstrated that EF are globally impaired in KS, KS patients performing poorly on shifting (Brokate et al., 2003; Fama et al., 2004), updating (Hildebrandt et al., 2004; Pitel et al., 2008), and inhibition (Fujiwara et al., 2008; Pitel et al., 2008). KS appears mostly associated with disinhibition, high interference sensitivity, poor judgment, and planning abilities, problem solving inabilities, and perseverative responses (Oscar-Berman, 2012). Moreover, explorations testing global dysexecutive symptoms (Van Oort and Kessels, 2009; Maharasingam et al., 2013) showed higher impairments in KS than ADS. However, very few studies directly compared ADS and KS performances (e.g., Brokate et al., 2003; Hildebrandt et al., 2004; Oscar-Berman et al., 2004; Pitel et al., 2008) and these investigations used tasks simultaneously involving several EF. Moreover, as each study focused on a limited range of tasks, the current data do not offer a comparison of the deficit across EF.

## What Do We Know about EF-Memory Interactions in KS?

Memory impairments are the key feature in KS, but their interactions with other cognitive functions are less explored. However, frontal lobes and EF play a critical role in memory performance, as illustrated by the prefrontal cortex involvement in memory (Habib et al., 2003; Salthouse et al., 2003; Parks et al., 2011; Kim et al., 2013). In the last three decades, there have been various attempts to establish reliable measures of EF among KS patients to understand their relation with memory (Squire, 1982; Leng and Parkin, 1988; Kopelman, 1991; Brokate et al., 2003). For example, by using a wide-range of frontal and memory tasks, Kopelman (1991) established an influence of frontal deficits on retrograde memory processes. In the same line, KS and post-encephalitic amnesic patients’ performance on WCST and Cognitive Estimation Test (CET) were compared (Leng and Parkin, 1988; Shoqeirat et al., 1990), showing a double dissociation for frontal dysfunction (i.e., impaired WCST but preserved CET performance for KS, and the reverse pattern for post-encephalitic amnesic patients). These results suggest that there are different patterns of frontal dysfunction that could be involved in memory deficit. More recently, some studies in KS confirmed that EF impairment is involved in memory deficits (Fama et al., 2004; Oscar-Berman, 2012). However, these previous studies were based on a correlational approach using separate explorations of EF and memory impairments, and not on the direct exploration of their interactions.

Interactions between EF and memory could also be illustrated by the context memory deficit hypothesis (Schnider et al., 1996) underlining the role of context memory impairment in amnesia. Indeed, KS patients are unable to remember background context such as spatio-temporal or intrinsic context (Borsutzky et al., 2008). In this view, context is regarded as “extra information” that can cue memory retrieval (Metcalf et al., 2007). Several studies (e.g., Kessels and Kopelman, 2012) have emphasized the relation between frontal lobe damage and contextual memory impairments, and disorientation-confabulation might follow memory and EF dysfunctions (Bouzerda-Wahlen et al., 2013). Therefore, the presence of spontaneous confabulation in KS arises from a combination of amnesia and frontal lobe dysfunctions (Kopelman, 1987). Although EF dysfunction is not univocally linked to frontal lobe damage, the crucial involvement of frontal regions in efficient executive abilities has been largely established during the last decades (Scott and Schoenberg, 2011). Frontal lobe damage might thus lead to executive control failure, which might impair the identification of events’ temporal context, those factors leading to repetitive confabulations (Kessels and Kopelman, 2012; Oscar-Berman, 2012). Centrally, spontaneous confabulations can be observed in KS (Borsutzky et al., 2008), resulting from strategic retrieval impairment, which results from executive dysfunction as they lead to search failure and wrong memories selection (Burgess and Shallice, 1996; Metcalf et al., 2007). Frontal lobes are thus clearly involved in spontaneous confabulations (Kopelman et al., 2009), which is further reinforced by neuroimaging studies showing that they are related to damage in ventro-medial and/or orbito-frontal regions (Kopelman et al., 2009). Indeed, while orbito-frontal damage does not necessarily result in EF dysfunctions, this area clearly belongs to a neural network involved in executive functioning (Szatkowska et al., 2007).

## What Should be Done in Future Studies?

A large literature has been developed on executive dysfunction in ADS (Stavro et al., 2013), notably showing the impact of executive dysfunctions on relapse (Bickel et al., 2012). Besides, studies on ADS investigated EF and their interactions with memory by using purer tests dedicated to one particular EF. For instance, ADS impairments in inhibition, shifting, and attentional bias for alcohol-related cues were observed during a go/no-go task (linked to inhibition performance) with neutral and alcohol-related words (Noël et al., 2005). Also, EF assessment (Fluency tasks, Stroop task, and n-back paradigm) revealed that performance on fluency task was a significant predictor of learning abilities (Spondee task, Pitel et al., 2007a). These results clearly demonstrate the implication of EF in some memory processes.

However, an accurate overview of executive functioning comparing ADS and KS cannot be established so far due to the lack of data and the use of composite executive tasks. Owing to the multifaceted nature of the EF tasks, it is hard to specify the contribution of each of Miyake’s factors (Shifting, Updating, and Inhibition) to some classic and/or complex tests routinely used to assess EF (Friedman and Miyake, 2004). The traditional EF tasks used for neuropsychological assessment are composite, resulting in a difficulty to investigate the continuum hypothesis for each EF. The flowchart in Figure 1A shows the EF overlap on certain tasks and underlines that most tests assessing EF in KS are multifaceted. Beside, since KS can be associated with varying nosological terms (i.e., ADS studies might have included a proportion of patients with undetected KS), an issue arises as to whether neuropsychological findings reflect various experimental design or differences in sample selection (Squire, 1982; Bowden, 1990). This perspective promote the importance of updating past neuropsychological findings (e.g., Leng and Parkin, 1988; Janowsky et al., 1989; Kopelman, 1991) on the relation between frontal dysfunction and memory impairment with a new experimental design program that includes a direct comparison between ADS and KS.

On the basis of studies presented above and questions held in abeyance, the possible line of approach for future studies will now be described by showing how EF may constitute a relevant research focus in the exploration of memory deficits in KS. This research approach is summarized in Figure 1B. Centrally, three research axes are proposed:

(1) Using specific EF tasks: future studies should use tasks evaluating each basic EF components (updating, shifting, and inhibition), e.g., the nine Miyake’s tasks (Miyake et al., 2000; see Figure 1B) which have been found to specifically explore the distinct EF subcomponents. The validity of these tasks has been reinforced by studies confirming the underlying multi-factorial model of EF in various healthy samples (Hedden and Yoon, 2006; Vaughan and Giovanello, 2010; Zheng et al., 2014). Although Miyake’s tasks have not yet been applied to psychiatric population, they are now widely used to explore EF (Fournier-Vicente et al., 2008) and present higher sensitivity and lower ceiling effect than classical EF tasks. In addition, a purer alternative to classic multifaceted planning tasks might be the unstructured task (Kramer et al., 2013) modeled after the six-elements test (Shallice and Burgess, 1991). This systematic exploration of EF subcomponents will clarify the differential deficit across EF in KS, identifying impaired and potentially preserved subcomponents.
(2) Systematically comparing ADS and KS: exploring and comparing thoroughly EF in KS, ADS, and healthy participants will step up the actual knowledge on this topic. Besides the nine tasks presented above, a specific exploration of inhibition subcomponent might be proposed (Friedman and Miyake, 2004; see Figure 1B), inhibition being classically considered as the central EF in addiction. This thorough comparison between ADS and KS will allow a direct testing of the validity of the continuum hypothesis in EF. It might indeed be postulated that the validity of this hypothesis varies across EF, a continuum being found for several EF subcomponents but not for others. In view of the massive inhibition impairments earlier observed in KS (Fujiwara et al., 2008; Pitel et al., 2008), it can be hypothesized that inhibition subcomponent will show a gradual decline from ADS to KS, a continuum not necessarily observed for other EF.
(3) Directly testing EF-memory interactions: the interaction between basic executive factors and retrieval memory process should be directly tested among KS and ADS. The Stop-Signal Task could be tailored to include a learning aspect based on classic memory learning task (e.g., California Verbal Learning Test; Elwood, 1995). Indeed, the Stop-Signal Task consists in two blocks of trials. During the first trial, participants have to perform a simple binary categorization task on words. Instructions for the second block are identical but participants have to inhibit their answer when hearing a sound after the word appearance. The modified Stop-Signal Task would include a preliminary block in which participants have to remember a list of words that will serve for the categorization (i.e., learned and non-learned words) in such a way that both inhibition and memory retrieval could be manipulated in the same task (i.e., inhibiting the prepotent response to the stop-signal while discriminating words). Contrary to classical approach (e.g., Shoqeirat et al., 1990) comparing results for separate memory and EF tasks, this method will simultaneously manipulate the two processes (inhibition and memory retrieval) in the same task to directly observe their interactions. More globally, the EF-memory interactions should be explored by innovative tasks combining specific EF subcomponent and memory demands.

To conclude, this perspective paper underlined the usefulness of developing more specific tasks to explore EF in KS. At the fundamental level, comparing ADS and KS performances with the above-mentioned tasks would clarify the debate on the continuum hypothesis by determining its validity for each EF and for EF-memory interactions. At the clinical level, a straightforward examination of EF process would identify the differential deficits in KS and lead to specific rehabilitation proposals already applied in ADS, e.g., cognitive enhancement (Sofuoglu et al., 2013) or inhibition training (Houben et al., 2011).

## Conflict of Interest Statement

The authors declare that the research was conducted in the absence of any commercial or financial relationships that could be construed as a potential conflict of interest.
